# Supplementary material for: Clinician Perspectives of Communication with Aboriginal and Torres Strait Islanders Managing Pain: Needs and Preferences
Source: Int J Environ Res Public Health. 2022 Jan 29;19(3):1572. doi: 10.3390/ijerph19031572 (PMC8835490; doi:10.3390/ijerph19031572)
Supplement: Supplementary file 1 [file ijerph-19-01572-s001.zip › Table S1 Cultural Awareness and Communication Needs Survey.pdf]

**Table S1.** Cultural Awareness and Communication Needs Survey

**Study ID** ____________ **Todays' date** ___ / ___ / ___

# - Yarning about Pain Study -

# Cultural Awareness and Communication Needs Survey

## Section 1. Recruitment details

1. **Study site**
   - ❑1 Princess Alexandra Hospital
   - ❑2 Townsville Hospital and Health Services
   - ❑3 Royal Brisbane and Women's Hospital

2. **What is your profession/area of work?**
   - ❑1 Pain specialist
   - ❑2 Registrar
   - ❑3 Clinical Nurse
   - ❑4 Physiotherapist
   - ❑5 Psychologist
   - ❑6 Pharmacist
   - ❑7 Aboriginal Health Worker
   - ❑8 Other, please specify:____________________________________________________

3. **What is your age (in years)?** ____________________________

4. **What is your sex?**
   - ❑1 Male
   - ❑2 Female
   - ❑3 Other

5. **Do you identify as Aboriginal or Torres Strait Islander?**
   - ❑1 Aboriginal
   - ❑2 Torres Strait Islander
   - ❑3 Both
   - ❑4 Neither

6. **Have you had previous cultural training?**
   - ❑1 No
   - ❑2 Yes

   If yes, please describe:____________________________________________________________

## Section 1. Communication skills and cultural awareness

1. **Please circle the rating that best represent your communication skills and knowledge while working with Aboriginal and/or Torres Strait Islander people with pain:**

| Items | Very low | Low | Moderate | High | Very high |
|---|---|---|---|---|---|
| **1.** How would you rate the **importance of communication training** for clinicians when working with Aboriginal and/or Torres Strait Islander patients | 1 | 2 | 3 | 4 | 5 |
| **2.** How would you rate your **knowledge** of how to effectively communicate with Aboriginal and/or Torres Strait Islander patients | 1 | 2 | 3 | 4 | 5 |
| **3.** How would you rate your **ability** to communicate with Aboriginal and/or Torres Strait Islander patients | 1 | 2 | 3 | 4 | 5 |
| **4.** How would you rate your **confidence** to communicate with Aboriginal and/or Torres Strait Islander patients | 1 | 2 | 3 | 4 | 5 |

***Thank you for completing this survey!***
