# Supplementary material for: Clinician Perspectives of Communication with Aboriginal and Torres Strait Islanders Managing Pain: Needs and Preferences
Source: Int J Environ Res Public Health. 2022 Jan 29;19(3):1572. doi: 10.3390/ijerph19031572 (PMC8835490; doi:10.3390/ijerph19031572)
Supplement: Supplementary file 1 [file ijerph-19-01572-s001.zip › Table S2 Script Focus group.pdf]

Table S2. Script for Focus Groups with Health Professionals

'***Clinical Yarning***: Improving communication and health outcomes for Aboriginal and Torres Strait Islander people living with chronic pain study'
Introduction: As outlined in the project information sheet, we are seeking to describe health professional's experiences with communication while supporting Indigenous patients diagnosed and treated for chronic pain. To do this, we would like to ask about your experience in treating and/or caring for Indigenous patients with chronic pain.

1. A good place to start is to describe your role and the patients in your care?

2. ***What is the patient usual path*** **to the pain clinic?** (Prompt: *Referral pathways? How much time patients stay in the waiting list?)*

3. ***Do you see Indigenous patients?*** *If not, why do think you do not see Indigenous patients?*

Next we would like to ask you about barriers and enablers to communication

4. ***What are the barriers/challenges when communicating with Aboriginal and Torres Strait Islander patients with persistent pain?***
Consider: Building rapport with the patient, gathering background information, forming a support plan or anything else.
Can you remember a situation where communication was complex or felt strange/uneasy to you?
When answering please be as specific as possible and provide examples from practice

5. Enablers to communication
What are the enablers/helps when communicating with Aboriginal and Torres Strait Islander patients with persistent pain?
Consider: Building rapport with the patient, gathering background information, forming a support plan or anything else.

6. ***How can training better improve communication?***
Consider: What format is most effective? How can training best be implemented to be accessible to you and your colleagues?
i) Mentorship/supervision
ii) How can cultural awareness training better enhance your practice, engagement or communication?
iii) What is the biggest challenge for you when communicating with Aboriginal and Torres Strait Islander patients?
iv) What health professional need to know about Aboriginal and Torres Strait Islander people and families to get better outcomes?
