# Supplementary material for: Clinician Perspectives of Communication with Aboriginal and Torres Strait Islanders Managing Pain: Needs and Preferences
Source: Int J Environ Res Public Health. 2022 Jan 29;19(3):1572. doi: 10.3390/ijerph19031572 (PMC8835490; doi:10.3390/ijerph19031572)
Supplement: Supplementary file 1 [file ijerph-19-01572-s001.zip › Table S3 Clinicians ratings.pdf]

Table S3. Comparison of Clinician's (*N*=64) Ratings of the Importance of Training, Knowledge, Ability and Confidence to Communicate with Indigenous Australians Patients Between Metropolitan and Regional Sites

Score	Combined Low 1-2				Moderate 3				Combined High 4-5				*p value*
Site	Metropolitan *n*=35		Regional *n*=29		Metropolitan *n*=35		Regional *n*=29		Metropolitan *n*=35		Regional *n*=29		
Items	*n*	(%)	*n*	(%)	*n*	(%)	*n*	(%)	*n*	(%)	*n*	(%)	
Perceived *importance of communication training* when working with Aboriginal and Torres Strait Islander patients	3	(9)	0	(-)	5	(14)	0	(-)	27	(77)	29	(100)	*0.019*
Perceived *knowledge* of how to effectively communicate with Aboriginal and Torres Strait Islander patients	10	(29)	1	(3)	17	(49)	18	(62)	8	(23)	10	(35)	*0.025*
Perceived *ability* to communicate with Aboriginal and Torres Strait Islander patients	4	(11)	0	(-)	26	(74)	18	(62)	5	(14)	11	(38)	*0.025*
Perceived *confidence* to communicate with Aboriginal and Torres Strait Islander patients	5	(14)	0	(-)	24	(69)	17	(59)	6	(17)	12	(41)	*0.019*
